# Psychological Health Status of Psychiatric Patients Living in Treatment Communities before and during the COVID-19 Lockdown: A Brief Report

**DOI:** 10.3390/ijerph18073567

**Published:** 2021-03-30

**Authors:** Pierluigi Cordellieri, Benedetta Barchielli, Valeria Masci, Francesca Viani, Ivan de Pinto, Andrea Priori, Felice Damiano Torriccelli, Chiara Cosmo, Stefano Ferracuti, Anna Maria Giannini, Jessica Burrai

**Affiliations:** 1 Department of Psychology, Sapienza University of Rome, Via dei Marsi 78, 00185 Rome, Italy; pierluigi.cordellieri@uniroma1.it (P.C.); annamaria.giannini@uniroma1.it (A.M.G.); 2 Department of Dynamic and Clinical Psychology, and Health Studies, Sapienza University of Rome, Via degli Apuli 1, 00185 Rome, Italy; benedetta.barchielli@gmail.com; 3 Association of Therapeutic Communities “Reverie”, Via Località Passo Cavallone 4, 00060 Capena, Italy; masci.valeria@gmail.com (V.M.); francescaviani93@gmail.com (F.V.); ivandp94@libero.it (I.d.P.); andreapriori7@gmail.com (A.P.); feldatorr@gmail.com (F.D.T.); 4 Department of Human Neuroscience, Sapienza University of Rome, Viale dell’Università 30, 00185 Rome, Italy; chiaracosmo@hotmail.it (C.C.); Stefano.ferracuti@uniroma1.it (S.F.)

**Keywords:** COVID-19, psychiatric patients, mental illness, cognitive function, psychiatric symptoms, risk perception, social support, lockdown

## Abstract

Many studies investigated the psychological impact of lockdown measures on the general population, while few studies focused on the psychiatric population. This study aimed to investigate the role of therapeutic communities in the management and containment of symptoms of patients with psychosis living in psychiatric residential facilities. Data were collected at two different points: November 2019 (Coronavirus disease 19 had not yet spread) and April 2020 (during the lockdown in Italy). Twenty-two study participants were recruited from three residential accredited psychiatric facilities. During lockdown, the patients showed a small increase in symptomatology in terms of emotional isolation. In addition, it was been observed significant differences in certain functional areas of the behavior, measured as lower inclination towards violent behaviors during lockdown, and higher scores in substance abuse and medical impairment. The lockdown condition could represent a form of containment; daily routines, along with adequate social support, are important aspects of the stability and the level of behavioral functioning of psychiatric patients. Social support and continuity of care offered by psychiatric communities can be an effective safeguard against the psychological impact of the COVID-19 epidemic.

## 1. Introduction

Residential facilities are a key resource for the Italian Mental Health Department; the facilities are dedicated to the treatment of patients suffering from mental illness who require therapeutic rehabilitation or social and health support interventions in residential settings. Nonmedical residential care facilities (RCFs) are a common residential setting for many people with mental illness, especially those with limited social support and greater supervision and care needs. Residential service models emerged as alternatives to deinstitutionalization, and RCFs base their work on the continuity of care; patients who moved back and forth between different care settings were most likely to change residence and to have the highest number of short admissions [1], while the continuity of the care setting could play a role in containment and help in the management of symptomatology [2]. 

Therapeutic psychiatric communities are complex organisms with a complex care path defined spatially and temporally. The path begins at the initial moment of reception in individual interventions, group interventions, or interventions with family members, and it is a path of inclusion, attachment, and detachment with involvement in the social network; great importance is given to the daily life and climate of the patient [3]. 

Indeed, residential programs in therapeutic psychiatric communities are often based on the integration of educational, psychiatric, and psychotherapeutic treatments within a therapeutic setting [4]. The assumptions of therapeutic psychiatric communities are represented by the shared construction of a therapeutic project between the patient, family, sending service, and community staff, and, moreover, by the therapeutic alliance that is built after a preliminary phase and that each community must try to guarantee [5,6]. 

Community-based residential mental health services are judged to be less restrictive and regimented models of care; for these reasons, they are considered less isolating and stigmatizing than other models of care [7]. Clinical intervention in the healthcare organization involves overcoming an individualistic conception [8] derived from the medical model, according to which the only patient is the individual. In addition, over time, increasing importance has been given to relational and intersubjective conceptions, highlighting the importance of social ties for the mental health of individuals and groups.

The group constitutes the modality through which the community of care can operate to achieve its aims [9]; that is, it is a space for the sharing and symbolic re-elaboration of experiences of suffering and the sharing of experiences, which are nourished by the transformative and “generative” capacity characteristic of “group thought” [10]. Despite growing evidence for their effectiveness, little research has been conducted to establish how therapeutic communities (TCs) work to produce positive outcomes. Pearce and Pickad [11] argued that there are two specific factors that, in combination, contribute to TC effectiveness: the promotion of a sense of belongingness and the capacity for responsible agency. Although both factors are found in other therapeutic approaches and are important to the psychosocial aspects of psychiatric care, the authors argued that their combination, extent, and emphasis are unique to TCs [11]. These characteristics could be considered crucial during the lockdown implemented to avoid the spread of coronavirus disease 19 (COVID-19). Patients living in psychiatric treatment communities during the COVID-19 lockdown showed unchanged depressive, anxious, and stressful symptoms; in particular, residential patients had lower perceived stress scores due to the COVID-19 situation compared to those of the general population, and the uninterrupted care provided by the residential community was considered to be an important protective factor [12]. In contrast, psychiatric patients, a population that could be considered at greater risk of distress and psychosocial pathological responses to exposure to a stressful situation such as a COVID-19 lockdown, were underinvestigated [13]. In people with preexisting mental illness, the impact of COVID-19 may be different than that for the general population. A rapid review of the literature on the potential impact of COVID-19 on psychotic patients during past epidemics and pandemics (e.g., Severe acute respiratory syndrome, SARS; Swine influenza, H1N1; Ebolavirus disease, EVD; Middle east respiratory syndrome coronavirus infection, MERS-CoV, and Equine influenza) highlighted that individuals with preexisting psychosis appeared to be less compliant with measures to prevent the spread of the virus (e.g., physical distancing and personal hygiene) [14]. Even in the healthy population, compliance factors are important in preventing the spread of the virus, although they are not often applied [15]. 

To the best of our knowledge, a comparison between symptomatology before and during the pandemic situation in the psychiatric population has not yet been performed. Aiming to address this gap, we compared clinical conditions of the psychiatric population living in health facilities before and during the COVID-19 pandemic in several domains, such as psychological impairment, social skills, and psychiatric symptoms. The study aimed to investigate the role of therapeutic communities in the management and containment of symptoms of patients with psychosis living in psychiatric residential facilities. The first data collection was conducted in November 2019 (COVID-19 had not yet spread), and the second was conducted in April 2020 (during the lockdown in Italy).

## 2. Materials and Methods

### 2.1. Participants

Twenty-two study participants were recruited from three residential accredited psychiatric facilities in Rome and Capena (Italy). These facilities are psychiatric communities that provide healthcare assistance through qualified personnel 24 h per day. Various professional figures work closely with psychiatric patients within the communities: psychologists, psychiatrists, educators, nurses, and social assistants. The therapeutic model of these communities evolved from the work of Wilfred Bion and John Rickman [16], and more generally from the first British therapeutic communities [17,18]. All patients carry out individual and group activities involving pharmacological, psychotherapeutic, rehabilitation, and socialization interventions. During the lockdown, all the professionals continued to work in the communities, guaranteeing the psychiatric patients’ continuity of care and treatment. Positive reinforcement techniques were used to encourage participation in therapy groups to prepare the patients to face social isolation and emotional flattening. 

All participants voluntarily responded to the anonymous survey and provided their informed consent. The sample included 12 males (54.5%) and 10 females aged between 19 and 45 years, with a mean age of 31.82 (SD = 6.69). The descriptive statistics and participant diagnoses are reported in Table 1. The exclusion criteria were (a) an inability to provide informed consent (i.e., Mini Mental State Examination < 8) and (b) a disease affecting the central nervous system (CNS). The study was approved by the Institutional Board of the Department of Human Neuroscience, Faculty of Medicine and Dentistry, “Sapienza” University of Rome (IRB-2020-6), in conformity with the principles of the Declaration of Helsinki. The descriptive statistics of the sample (Table 1) are reported.

### 2.2. Procedures

The first data collection (T1) was conducted in November 2019 (non-COVID time, hereinafter NoCoT). The patients were evaluated using the following clinical scales: the Mini Mental State Examination (MMSE), Brief Psychiatric Rating Scale (BPRS), and Kennedy Axis V (K Axis). The second data collection (T2) was conducted in April 2020 (COVID time, CoT). The patients were evaluated with the same scales as those at T1, but specific items were added on COVID-19 to investigate the psychiatric patients’ knowledge and risk perception about the COVID-19 pandemic. Information on COVID-19 was collected through self-report items (i.e., “How did you become aware of the spread of COVID-19?”; “What is COVID-19?”; “Did you participate in community training sessions on this health emergency?”).

### 2.3. Materials

Sociodemographic information was collected with a questionnaire developed ad hoc that included items on gender, age, marital status, education level, substance use, socioeconomic status, psychiatric diagnosis, presence of any other pathology, time spent in the community, and relationships with family.

Validated and reliable measures were used to assess the patients’ cognitive functions, psychiatric symptoms, and several specific areas of functioning. Cognitive domains (orientation to time and space, registration of three words, attention, and calculation, recall of three words, language, and visual construction) were measured with the MMSE [19]. Psychiatric symptoms were measured with the 24-item Brief Psychiatric Rating Scale [20]. The K Axis [21] was used to measure the patients’ overall functioning and functioning in several specific areas, with each area of functioning scored on a continuum of 100 points according to a decreasing order of severity (0 = very severe compression; 100 = high function). The investigated areas were as follows: (1) Psychological impairment; (2) Social skills; (3) Violence; (4) ADL-Occupational skills (5) Substance abuse; (6) Compromising of physical conditions: Medical impairment; (7) Ancillary impairment (legal, financial, milieu).

COVID-19 risk perception was measured with three items adapted from Cho and Lee [22]. Five items evaluated negative mood due to restrictive measures carried out in the community. 

For social support, the shorter version of the Multidimensional Scale of Perceived Social Support (MSPSS) [23] was used. The short version used in the present research contains three items ranked from 1 (not agree at all) to 7 (agree at all).

### 2.4. Statistical Analyses

ANOVA with repeated measures was used to compare the scores of the clinical scales in two different periods (NoCot vs. Cot). Statistical analyses were performed using Statistical Package for Social Science (SPSS; version 25.0; IBM SPSS, Armonk, NY, USA). In the pairwise comparisons, the Bonferroni correction for alpha inflation was performed.

## 3. Results

The descriptive statistics of the sample (Table 1), risk perception, negative mood, and social support (Table 2) are reported.

As shown in Table 3, we did not find statistically significant differences between the BPRS scores measured in November (T1; NoCoT) and April (T2; CoT). During lockdown, the patients showed a small increase in symptomatology (T1_NoCoT_ M = 2.50, T2CoT M = 2.79) in terms of emotional isolation, but differences in other symptoms were not found. The MMSE also did not show a significant difference (Table 3).

Otherwise, the K Axis scores showed a significant main effect (F _(1,6)_ = 9.996, *p* < 0.001; η_p_^2^ = 0.0435) and significant interaction effect of K Axis*Session (F _(1,12)_ = 3.157, *p* < 0.01; η_p_^2^ = 0.195). We observed significant differences in certain functional areas of the behavior measured by the K Axis between the two time points (Table 3). The comparisons revealed significant differences for violence (Area 3). Pairwise comparisons showed that the mean of T1 was lower (M = −13.333, SE = 0.09, *p* < 0.05) compared to the T2. Specifically, the patients showed a lower inclination towards violent behaviors during lockdown (higher scores indicate a lower level of criticality in this area).

A significant difference was also observed for area 5, substance abuse. Pairwise comparisons showed higher mean difference in the T2 compared to the T1 (M = −4.861, SE = 0.265, *p* < 0.05). This functional area seemed to improve during the lockdown (higher scores indicate a lower level of criticality in this area).

Finally, there were significant differences for medical impairment (Area 6). Pairwise comparisons showed a significant higher mean difference in the T2 compared with T1 (M = −7.917, SE = 0.004, *p* < 0.05). The physical condition of the patients improved during the lockdown (higher scores indicate a lower level of criticality in this area). No gender differences were found for any dimensions assessed. 

## 4. Discussion

Several recent studies have demonstrated a significant impact of the COVID-19 pandemic on psychological health, particularly as a result of the lockdown [24,25,26], but few studies have investigated this impact on specific populations, such as psychiatric patients.

The present study compared the psychiatric symptoms and functioning in several specific areas of patients living in residential communities before and during the lockdown in Italy. An important result emerged from the comparison between the clinical evaluations from November 2019 (before the lockdown in Italy) and April 2020 (during the lockdown in Italy). According to our data, the patients did not show an increase in psychiatric symptoms; the only exception was a small increase in emotional isolation. The increased feeling of emotional isolation may have been linked to the isolation imposed by the necessary containment of COVID-19. Although social isolation is part of the symptomatology of many psychiatric disorders [27], the limitations imposed during the lockdown may have exacerbated the sense of loneliness and despair due to the imposed distance from loved ones but also staff and other psychiatric patients in the community. In contrast, different functional areas of behavior showed improvements: there was a lower propensity for violent behaviors, lower rates of substance abuse, and better physical conditions.

These findings may seem to contrast with those of numerous studies that have indicated concerns about the pandemic or reported that a period of isolation can lead to an increase in psychopathologies, including psychotic psychopathologies [28]. Systematic reviews and specific studies have shown significant effects of the COVID-19 pandemic on the psychiatric population [29,30,31]. Forced quarantine to combat the spread of COVID-19 has produced forms of acute panic, anxiety, obsessive behavior, paranoia, and depression in psychiatric patients. 

In the same studies, however, it was recognized that acute pathological conditions increase with concomitant causes of stressors, such as psychological vulnerability, social isolation, unemployment, relational rupture, etc. In particular, social isolation seems to be the variable that “carries the most weight” for the psychiatric population. For example, Giallonardo and colleagues [32] showed that if protracted, social isolation may increase the risk of recurrences of episodes of mental disorders beyond triggering the onset of new mental disorders in the most vulnerable people. Moreover, objective social isolation and subjective feelings of loneliness are associated with a higher risk of suicidal ideation and suicide attempts. For many persons with mental disorders, being alone is a heavy burden, far greater than that experienced by many other persons. Moesmann and colleagues [27] reported that in their nonresidential clinics, some patients went from a high level of functioning to a need for hospitalization due to the rupture of their weekly routines. In some cases, telepsychiatry and other cutting-edge technologies have been effective tools in bridging social distance and ensuring continuity in mental health assistance [33].

Research has shown the importance of ensuring social support and mental health care for patients with mental disorders [34]. In the literature, differences between psychiatric outpatients and inpatients have been reported. Outpatients have been shown to experience greater psychological impact on their mental health, with higher depression, anxiety, and stress scores than healthy controls [29,35] due to the interruption of some psychiatric services and the difficulties accessing these services due to the lockdown. Therefore, continuous monitoring of the medical and psychological health of patients receiving mental health services is essential to design and respond to problems arising from the lockdown and the spread of the virus [36]. On the other hand, inpatients have been found to experience greater confidence in being protected from virus than control groups, as they feel protected by hospital staff [37]. However, inpatient psychiatric settings have faced new challenges: close contact between staff and patients, the restriction of visitors, and the recommendation of improved hygiene [38].

In our study, the subjects were residential patients in therapeutic communities and were therefore protected from different social stressors, such as relational continuity and low exposure to mass and/or social media. During quarantine, the patients’ days were spent engaging in routine activities. Twice a week, the patients could call their families to ensure their health. The peer group or community psychologists provided ongoing social support. Therefore, we believe that the patients in our study did not have worsening symptoms due to the continuity of social support and medical care. 

We observed that some functional areas of behavior improved. These behavioral areas were mainly linked with containment aspects [39]. “Containment” is a broader term that includes a wide variety of strategies, including pharmacological treatment and nonpharmacological interventions or techniques, such as increased observation levels, locked wards, de-escalation techniques, the use of behavioral agreements and increased staffing levels. In this study, we refer to the conditions imposed due to COVID-19 outbreak: an inability for patients to leave the community, the use of only telephone meetings with family and friends, etc. Paradoxically, for the patients in our study, these measures likely resulted in less exposure to social stressors. Indeed, the family environment can either play a protective and detrimental role [40] and for psychiatric patients, not being embedded in dysfunctional family dynamics (e.g., low family cohesion and low caregiver warmth) may have contributed to a stability in symptom severity.

Our hypothesis is that the lockdown condition represented a further form of containment. Daily routines, along with adequate social support, are important aspects of the stability and the level of behavioral functioning of psychiatric patients, in particular for those with anxiety, violent acts, and substance abuse. In summary, we believe that social support and continuity of care offered by psychiatric communities can be an effective safeguard against the psychological impact of the COVID-19 epidemic.

We are aware of the limitations of our research. The limited number of subjects could not ensure the external validity of our research. In addition, our investigation involved patients from a single community association. It could also be very interesting to extend our results to other residential contexts. We believe, however, that our results provide interesting insight and may be a stimulus for further research on the severe psychiatric patient population during COVID-19 and in directing further research on patients living in treatment communities. 

## 5. Conclusions

Lockdown measures are still the best available containment strategy in limiting the spread of viruses despite their negative long-lasting psychological impact related to isolation and loneliness.

The impact of COVID-19 may differ from the general population in psychiatry patients; however, the responses to exposure to a stressful situation, such as a COVID-19 lockdown, in psychiatric patients have been underinvestigated.

The present study compared the psychiatric symptoms and functioning in several specific areas of patients living in residential communities before and during the lock-down in Italy. Lockdown measure may be an additional form of containment along with daily routines and adequate social support that can be an effective safeguard against the psychological impact of the COVID-19 outbreak. 

## Figures and Tables

**Table 1:** Descriptive statistics of the study sample.

Characteristic	Group	Psychiatric Patients *N* (%) = 22
Age	*M* (*SD*)	31.82 (6.96)
	Min–Max	19–45
Gender	Female	10 (45.5%) 12 (54.5%)
	Male	
Education	Middle school diploma	8 (36.4%)
	High school diploma	12 (54.5%)
	Graduate	2 (9.1%)
Diagnostic Criteria	Schizophrenia Delusional Disorder Schizoaffective Disorder Depressive Disorders Bipolar and Related Disorders Personality Disorders	6 5 5 1 1 4

**Table 2:** Descriptive statistic of COVID information and risk perception, bad mood, and social support during the quarantine.

Dimension	M (*SD*) Min-Max	Cronbach’s Alpha (*a*)	*N* (%)
Participation informative meetings about COVID-19	Yes		22 (100%)
Risk perception	11.87 (2.27)	0.725	22 (100%)
	3–15		
Bad mood due to restrictive measures	16.06 (3.68)	0.72	22 (100%)
	5–25		
Social support	14.56 (4.95)	0.818	22 (100%)
	3–21		

**Table 3:** Between administration-time differences (ANOVA).

Clinical Scale							
Dimension	F	*p*	η_p_^2^	Multiple Comparisons	Mean Difference	Std. Error	Sig.
MMSE	1.56	0.234	0.107	T1 vs. T2			
BPRS	0.296	0.596	0.022	T1 vs. T2			
K_Axis	3.157	0.008	0.195	T1 vs. T2			
PI				T1 vs. T2	1.25	1.821	-
SS				T1 vs. T2	3.194	3.051	-
Vi				T1 vs. T2	−13.333	5.090	0.05 *
OI				T1 vs. T2	−5.556	0.021	-
SA				T1 vs. T2	−4.861	0.265	0.05 *
CPC				T1 vs. T2	−7.917	0.004	0.01 **
AI				T1 vs. T2	0.000	1.00	-
GAF Eq.	4.316	0.058	0.249	T1 vs. T2			
GAF K	0.671	0.428	0.049	T1 vs. T2			
DL	0.985	0.339	0.070	T1 vs. T2			

* *p* < 0.05. ** *p* < 0.01; K_Axis = Kennedy Axis V; PI = Psychological impairment; SS = Social skills; Vi = Violence; OI = ADL-Occupational Skills; SA = Substance abuse; CPC = Compromising of physical conditions: Medical impairment; AI = Ancillary impairment; GAF Eq = Global Evaluation Functioning Equivalent, a score that provides an average and global representation of the patient’s functioning. It is obtained from the average of the first four Kennedy Axis V scales; GAF K = Global assessment of functioning. Global functioning obtained by selecting the lowest of the scores from the first four areas; DL = Danger level, this index identifies the highest risk score among those obtained in the seven areas.

## Data Availability

The data presented in this study are available on request from the corresponding author. The data are not publicly available due to request from Mental Community where data have been collected.
